# Positive Influence of a Probiotic Mixture on the Intestinal Morphology and Microbiota of Farmed Guinea Fowls (*Numida meleagris*)

**DOI:** 10.3389/fvets.2021.743899

**Published:** 2021-10-29

**Authors:** Livio Galosi, Salvatore Desantis, Alessandra Roncarati, Patrizia Robino, Alessandro Bellato, Patrizia Nebbia, Ilario Ferrocino, Nicoletta Santamaria, Lucia Biagini, Lorenzo Filoni, Anna Rita Attili, Giacomo Rossi

**Affiliations:** ^1^School of Biosciences and Veterinary Medicine, University of Camerino, Matelica, Italy; ^2^Department of Emergency and Organ Trasplants (DETO), University of Bari Aldo Moro, Valenzano, Italy; ^3^Department of Veterinary Sciences, University of Torino, Grugliasco, Italy; ^4^Department of Agriculture, Forestry and Food Science, University of Torino, Grugliasco, Italy

**Keywords:** guinea fowl, *Numida meleagris*, Slab51®, intestinal morphology, intestinal microbiota

## Abstract

To understand the effectiveness of a probiotic mixture on intestinal morphology, mucus layer composition, and cecal microbiota diversity, 40 10-day-old Guinea fowls (*Numida meleagris*) were assigned to two groups: the control group (C), receiving drinking water, and the treated group (P), receiving water plus a commercial multi-strain probiotic (Slab51®, 2 × 10^11^ CFU/L). Birds were slaughtered after 4 months, and the intestines were collected. Samples from the duodenum, ileum, and cecum were processed for morphological and morphometric studies, and conventional glycohistochemistry. Cecal samples were also used to assess the microbiota by 16S metataxonomic approach. Group P showed significant increase in the villus height (*p* < 0.001 in the duodenum and *p* < 0.05 in the ileum and cecum), villus width (*p* < 0.05 in all investigated tracts), depth of crypts (*p* < 0.001 in the duodenum and cecum; *p* < 0.05 in the ileum), and goblet cells per villus (*p* < 0.001 in all investigated tracts) compared with group C. Cecal microbiota of the birds varied considerably and comparing the relative abundance of the main observational taxonomic units (OTUs), a positive enrichment of several beneficial taxa, such as *Oscillospira, Eubacterium, Prevotella*, and members of the *Ruminococcaceae*, was observed. The enrichment of those taxa can improve microbiota stability and resilience facing environmental stresses, enhancing its resistance against invading pathogens. *Ruminococcaceae*, which represent the most important taxon in both groups, and *Prevotella* have a key role in the gut physiology due to the production of short-chain fatty acids (SCFAs), which are a vital energy source for enterocytes, improve glucose metabolism, and exert an overall anti-inflammatory effect. Probiotic administration enriches the presence of *Coprococcus, Oscillospira*, and *Eubacterium* taxa that produce butyrate, which exerts a beneficial effect on growth performance, structure of villi, and pathogen control and has anti-inflammatory properties too. This study indicates that Slab51® supplementation positively affects the morphology and microbiota diversity of the guinea fowl intestine.

## Introduction

Considering the increasing consumer demand for “natural” products, and the ban of antibiotics in livestock and poultry breeding in many countries (1), the use of probiotics has increasingly been considered a profitable opportunity to obtain mutual benefit for both consumers and industries and for poultry well-being (2).

Probiotics are live microorganisms, which, administered in adequate amounts, have a positive effect on the health and growth efficiency of the host by influencing gut microbiota or modifying immune status, as well as by stimulating digestive processes (2–5). Formerly, probiotics used to be *Lactobacillus* spp. and *Bifidobacterium* spp., mostly selected for their capability to survive gastrointestinal conditions, and adhere to epithelial cells (6, 7). Many authors focused on the specific actions that the individual strains composing probiotic mixtures have (8, 9). On the other hand, it is essential to denote that, thanks to the variation in inhibitory mechanisms, mixed-culture probiotics appear to be more effective in inhibiting pathogens than single-strain probiotics (10). The importance of the intestinal microbiota for gastrointestinal function and health have been reported in both mammalian (11) and avian species (2, 12, 13). Regarding the avian species, many studies have demonstrated the effects of dietary probiotic administration in modifying the intestinal gene expression (14), physiology (15), immunology (15, 16), morphology, and mucus composition (12, 14–18). The above-cited investigations indicate that the effects on gut morphology and mucins depend on probiotic composition. The production of mucins takes place in the goblet cells (GCs), and the mucin transcription is regulated by a variety of bioactive factors, including microbiota and its secretory products (11, 19). Mucins are glycoproteins that play a key role in constituting the mucus layer covering the epithelium of the gastrointestinal mucosa. The mucus layer protects the epithelium against physical and chemical injuries caused by food, microbes, and microbial metabolites; promote the gut content elimination (11, 19); and modulate water and electrolyte absorption (20). Interestingly, the intestinal commensal microbiota depends on mucus and undigested dietary carbohydrates for binding sites and energy sources; moreover, the intestinal microbiota affects the functions of the intestinal epithelium, including those of the GC and mucus layers, by a “cross-talk” feedback mechanism (11, 21). Guinea fowls (*Numida meleagris*) represent a new promising species as source of poultry meat for human consumption. However, its low meat production and high feed conversion rate (FCR) result in low yield and high breeding cost (22). Therefore, several feeding strategies are being tested to improve the performance, growth, and meat production (23). Recently, it has been shown that the dietary supplementation with a commercial multi-strain probiotic (Slab51®, Ormendes SA, Jouxtens-Mézery, CH) containing a mixture of different species of lactic acid bacteria and bifidobacteria induced enhancement of growth performance and changes in the composition of mucins in the intestinal tract of pigs (24, 25) as well as clinical amelioration in dogs and cats with chronic intestinal pathology (26, 27).

Especially, *Lactobacillus acidophilus* is reported to have a negative effect on bacterial cell proliferation, migration, and invasion capacity. Furthermore, many studies confirmed that *Lactobacillus* spp. can modulate the composition of the intestinal microbiota, and its activity, resulting in an enhanced epithelial function and improved intestinal health. *Streptococcus termophilus* activity on the host health is supposed to be mediated by different mechanisms such as the production of thermophilins, able to reduce the invasion by pathogens. *In vitro, S. termophilus* has manifested antioxidant and immunomodulation activities, which can both contribute to intestinal health (9). One of the major benefits of *Bifidobacterium lactis* is decreasing a leaky gut by lowering the permeability of the gut wall and stopping foreign harmful substances from passing into the body (28).

On this basis, the aim of this study was to evaluate the effect of the above-cited probiotic complex on the intestinal morphology, goblet cell number and the related mucin production, and cecum microbiota of guinea fowls.

## Materials and Methods

### Animal Trials

At 10 days of age, 40 unsexed healthy pearl guinea fowls (*Numida meleagris*), with an average weight of 110.00 ± 0.99 g, were randomly assigned to two groups: the control group (C) received water without any additive, while the treated group (P) received water supplemented with the probiotic mixture Slab51®, at a dosage of 2 × 10^11^ colony-forming units per liter (CFU/L). The water was given *ad libitum* to each fowl to ensure water intake and to reproduce the usual way of administration used in fowl rearing. Water intake varies greatly based on environmental factors (e.g., temperature and relative humidity), feed composition, calendar period, and the age of the birds. Slab51® (recently marketed in Europe under the trademark SivoMixx®, Ormendes SA, Jouxtens-Mezery, CH) is a commercial multi-strain probiotic containing 200 billion lactic acid bacteria per 1.5 g of product, comprised of the following strains: *Streptococcus thermophilus* DSM 32245/CNCM I-5570, *Bifidobacterium lactis* DSM 32246/CNCM I-5571, *Bifidobacterium lactis* DSM 32247/CNCM I-5572, *Lactobacillus acidophilus* DSM 32241/CNCM I-5567, *Lactobacillus helveticus* DSM 32242/CNCM I-5573, *Lactobacillus paracasei* DSM 32243/CNCM I-5568, *Lactobacillus plantarum* DSM 32244/CNCM I-5569, and *Lactobacillus brevis* DSM 27961/CNCM I-5566. Both groups were housed in a commercial farm, in two adjacent sheds (12 m^2^ each), with litter on the bottom, under controlled photoperiod (days 1–28: 23-h L/8-h D; days 29–120: natural according to summer season), and natural aeration. Throughout the trial, both the groups received the same commercial pellet feed, administered *ad libitum*, as starter feed (Broilers Gialli 1 0/22 BR, Mangimi Cruciani Srl, Montappone, MC, Italy) followed by growing feed (Broilers Gialli 2 22/42 BR, Mangimi Cruciani Srl, Montappone, MC, Italy), that changed in proximate composition in relation to the age of the animals (Table 1). At the end of the normal growth process, at 120 days of age, birds of both the groups were weighted with an electronic balance (ACS-A9, My Scale, Foggia, Italy), before being slaughtered by electrical stunning and bleeding.

**Table 1:** Proximate composition of the feeds administered to both the groups of guinea fowls (*Numida meleagris*) during the trial.

	***Starter***	***Grower***
**Feeding phases of guinea fowls (days)**	**10–40**	**41–120**
**Proximate composition (%)**	
Protein	22.0	21.0
Lipids	5.0	5.8
Ash	7.4	7.5
Fiber	4.4	4.5
Calcium	1.00	1.04
Phosphorum	1.00	0.85
Sodium	0.20	0.19
Lysine	1.2	1.2
Methionine	0.60	0.58
Phytasis (FTY)	1,500	750
Endo-1.4-beta-xylanasis (FXU)	200	–
Vitamin A E672 (IU/kg)	12,000	4,000
Vitamin D3 E671 (IU/kg)	2,000	1,250
Vitamin E (91% alpha-tocopherol) (mg/kg)	40	20
Copper E4 (mg/kg)	16	10
Selenium E8 (mg/kg)	0.16	0.20
Lutein E161b (g/kg)	–	41
Zeaxanthin E161 (g/kg)	–	8.4

From all the birds, segments of ~3 cm were collected from the duodenum, ileum, and one of the two cecum intestines and fixed in 4% (v/v) phosphate-buffered saline paraformaldehyde for 24 h at 4°C. The samples were then dehydrated through a graded series of ethanol and embedded in paraffin wax. Serial sections (5 μm thick) were cut, and after being dewaxed with xylene and hydrated in an ethanol series of descending concentrations, they were stained with hematoxylin–eosin for morphological studies and by histochemical procedures for mucin characterization.

The presence of GCs was demonstrated by alcian blue pH 2.5 (AB 2.5) and periodic acid–Schiff (PAS) staining sequence, which reveals acidic, neutral, and mixed mucins (29).

The second cecum intestine of the birds was collected immediately after evisceration, individually packed in plastic tubes and soon frozen at −80°C, until use for molecular test.

### Morphometric Measurement

Hematoxylin–eosin-stained sections of 10 well-oriented villi of the duodenum, ileum, and cecum from each animal were photographed with a ×4 lens using a light microscope (Eclipse Ni-U; Nikon, Japan) and used to measure the villus height (VH), villus width (VW), and the crypt depth (CD). Then, the ratio of the villus height to crypt depth (VH:CD) was calculated. In addition, the total number of GCs stained with AB 2.5–PAS method was determined by counting both sides of the 10 well-oriented villi of the duodenum, ileum, and cecum tracts with a ×10 lens. The density of GCs as the number per 100 μm of villus length (15, 18) was also counted. Images were analyzed with the image-analyzing program NIS Elements BR (Version 4.30) (Nikon, JP).

### DNA Extraction and 16S rRNA High-Throughput Amplicon Target Sequencing

Total microbial DNA were extracted from cecal content using a commercial kit (QIAamp® DNA Microbiome, Qiagen, Hilden, Germany), according to the recommendations of the manufacturer. Briefly, the cecum was flamed, held flat with two surgical tweezers and cut along the longitudinal axis with a scalpel blade. Then a sterile swab was rubbed on the bottom of the cecal wall. Sterile instruments, DNA-free pipette tips, and consumables were used to avoid false results due to contamination. Eluted nucleic acids were quantified by NanoDrop instrument (Celbio, Milan, Italy) and DNA samples were standardized at 50 ng/μl and stored frozen (−20°C) until use. DNA extracts were used as template in the PCR amplifying the V3–V4 region of the 16S rRNA gene using the universal primers and protocols described by Klindworth et al. (30). PCR amplicons were cleaned and tagged according to the Illumina 16S metagenomic sequencing library preparation guidelines. Sequencing was performed with a MiSeq Illumina instrument that generated 250-bp paired-end reads according to the instruction of the manufacturer.

### Bioinformatics and Statistical Analysis

We set the maximum acceptable probability at 5% for type I error (α = 0.05) and at 20% for type II (β = 0.2). Based on previous studies, we expected a moderate to high magnitude of the probiotic effect on histologic measures (standardized range ≥0.9 standard deviations), also because to be relevant in terms of zootechnical practice, a probiotic would have to exert such an effect as to make the expenditure for farmers acceptable. Accordingly, the sample size was calculated to have 0.8 power of the study, 0.05 significance level, and 0.9 magnitude of effect by using the G^*^Power version 3.1.9.6 (31).

Morphometric measures are expressed as means ± standard deviation (SD). Differences in means were compared by multiple Student's *t*-test. The results were evaluated for statistical significance, and *p*-values are provided. To account for the probability of false positives due to multiple tests, the q-values were calculated as the estimate of the *a posteriori* probability of the null hypothesis for each test (32); *q*-values are reported along with *p*-values for each morphometric comparison.

After sequencing, raw reads were analyzed by the pipeline as previously reported (33). After using FLASH software for the assembly, QIIME 1.9.0 software was used for quality filtering, to assign taxonomy using the greengenes database, and to produce the OTU table. In order to avoid biases due to the different sequencing depth, OTU tables were rarefied at 7,720 sequences/sample. The OTU table displays genus level or family level. Diversity indices (alpha) were calculated using the *diversity* function of the *vegan* package in R (34). ANOSIM and ADONIS statistical test were used to find differences in microbial composition. Wilcoxon rank-sum tests were used as appropriate to determine significant differences in alpha diversity or OTU abundance. PICRUSt tools (35) were used to predict the potential metabolic pathway of the cecal microbiota. Not normally distributed variables were presented as median and box plots representing the interquartile range between the first and the third quartile, with the error bars showing the lowest and the highest value. Pairwise Spearman's non-parametric correlations were used to study the relationships between the relative abundance of microbiota and inferred metabolic pathway. The correlation plots were visualized in R using the *corrplot* package. A *p*-value of 0.05 or lower was considered as statistically significant.

### Nucleotide Sequence Accession Number

All the sequencing data were deposited in the sequence read archive of the National Center for Biotechnology Information under the BioProject number PRJNA630376.

## Results

All animals survived the experimental period (120 days). At the end of the trial, the probiotic group showed a final mean body weight (1,820.45 ± 209.00 g) higher than that of the control group (1,754.05 ± 140.00 g), but the effect was not large enough to be perceived with this sample size (*p* = 0.245).

### Morphometry

**Figure 1:**
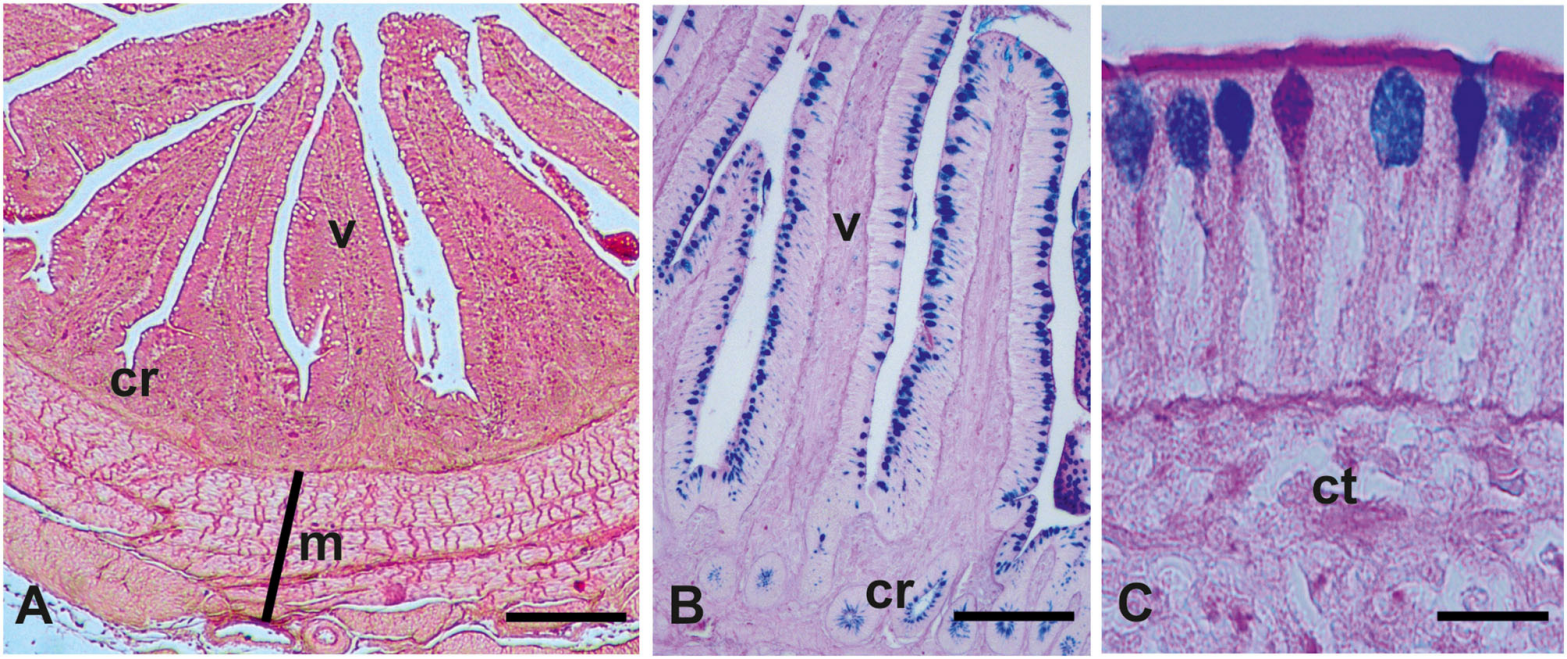
Representative histological **(A)** and glicohistochemical **(B,C)** views of the guinea fowls (*Numida meleagris*) intestine. cr, crypts; ct, connective tissue; m, muscularis; v, villum. **(A)** Hematoxylin–eosin staining; **(B,C)** Alcian blue pH 2.5 (AB)/periodic acid–Schiff (PAS) staining. **(C)** Picture shows goblet cells producing acidic mucins (azur staining, AB positivity), neutral mucins (magenta staining, PAS positivity), and both acidic and neutral mucins (violet staining, AB/PAS positivity). Scale bars **(A)** 250 μm, **(B)** 200 μm, and **(C)** 15 μm.

The intestinal mucosa consisted of villi, finger-like projections, and basal crypts (Figures 1A,B). Villi were covered by a simple striated columnar epithelium and GCs were distributed among columnar cells (Figures 1B,C). The results of intestinal morphometric analysis of both the control (C) fowls and probiotic-treated (P) fowls are displayed in Table 2. Probiotic supplementation induced significant increase of VH (*p* < 0.001 in the duodenum and *p* < 0.05 in the ileum and cecum), VW (*p* < 0.05 in all investigated tracts), and CD (*p* < 0.001 in the duodenum and cecum; *p* < 0.05 in the ileum) compared with controls. The significant difference in VH observed in the cecum (*p* < 0.05) was potentially a false positive based on the adjustment of the *p*-value for multiple comparisons (*q* > 0.05). No statistical difference was observed in VH:CD between the P and C groups. AB 2.5/PAS staining, which detects all types of acidic mucins and neutral mucins at the same time, revealed that the number of GCs per villus was statistically increased (*p* < 0.001) in the intestine of the P animals, whereas the density of GCs (cell numbers per 100 μm of villus length) was not statistically different between the C and P fowls (Table 2). The histochemical investigation revealed that most GCs of the intestinal villi from both C and P produced acidic mucins (AB2.5 positivity), whereas a few GCs produced neutral mucins (PAS positivity) (Figures 1B,C).

**Table 2:** Effects of the multi-strain probiotic Slab51® supplementation on the intestinal morphology of two guinea fowls (*Numida meleagris*) groups (C and P) (mean ± standard deviation).

**Item**	**Control (C)**	**Probiotic (P)**	***p*-value**	***q*-value**
**Duodenum**				
Villus height (μm)	649.11, 140.22	895.71, 122.13	<0.001	<0.001
Villus width (μm)	107.48, 20.55	132.14, 22.10	0.025	0.041
Crypt depth (μm)	101.58, 23.07	143.38, 27.46	<0.001	<0.001
Villus height:crypt depth	6.71, 2.24	6.57, 1.31	0.728	0.771
Goblet cell numbers/100 μm	9.47, 3.02	9.44, 2.1	0.969	0.969
Goblet cell numbers per villus	122.92, 7.62	169.11, 14.32	<0.001	<0.001
**Ileum**				
Villus height (μm)	671.88, 88.69	747.52, 156.18	0.010	0.023
Villus width (μm)	130.30, 17.12	156.13, 31.95	0.023	0.041
Crypt depth (μm)	110.97, 16.8093	126.58, 25.108	0.002	0.005
Villus height:crypt depth	6.33, 1.34	6.00, 1.42	0.129	0.155
Goblet cell numbers/100 μm	9.65, 2.25	9.60, 2.29	0.463	0.112
Goblet cell numbers per villus	139.24, 11.98	155.73, 3.27	<0.001	<0.001
**Cecum**				
Villus height (μm)	464.81, 232.47	594.97, 268.88	0.043	0.065
Villus width (μm)	140.41, 25.53	162.45, 23.16	0.023	0.041
Crypt depth (μm)	84.82, 21.54	119.10, 29.86	<0.001	0.001
Villus height:crypt depth	5.47, 4.3	4.99, 3.03	0.087	0.112
Goblet cell numbers/100 μm	9.88, 4.06	8.43, 3.48	0.083	0.112
Goblet cell numbers per villus	94.09, 4.76	141.15, 13.81	<0.001	<0.001

*The statistical significance was set at p < 0.05*.

### Cecal Microbiota Characterization

**Figure 2:**
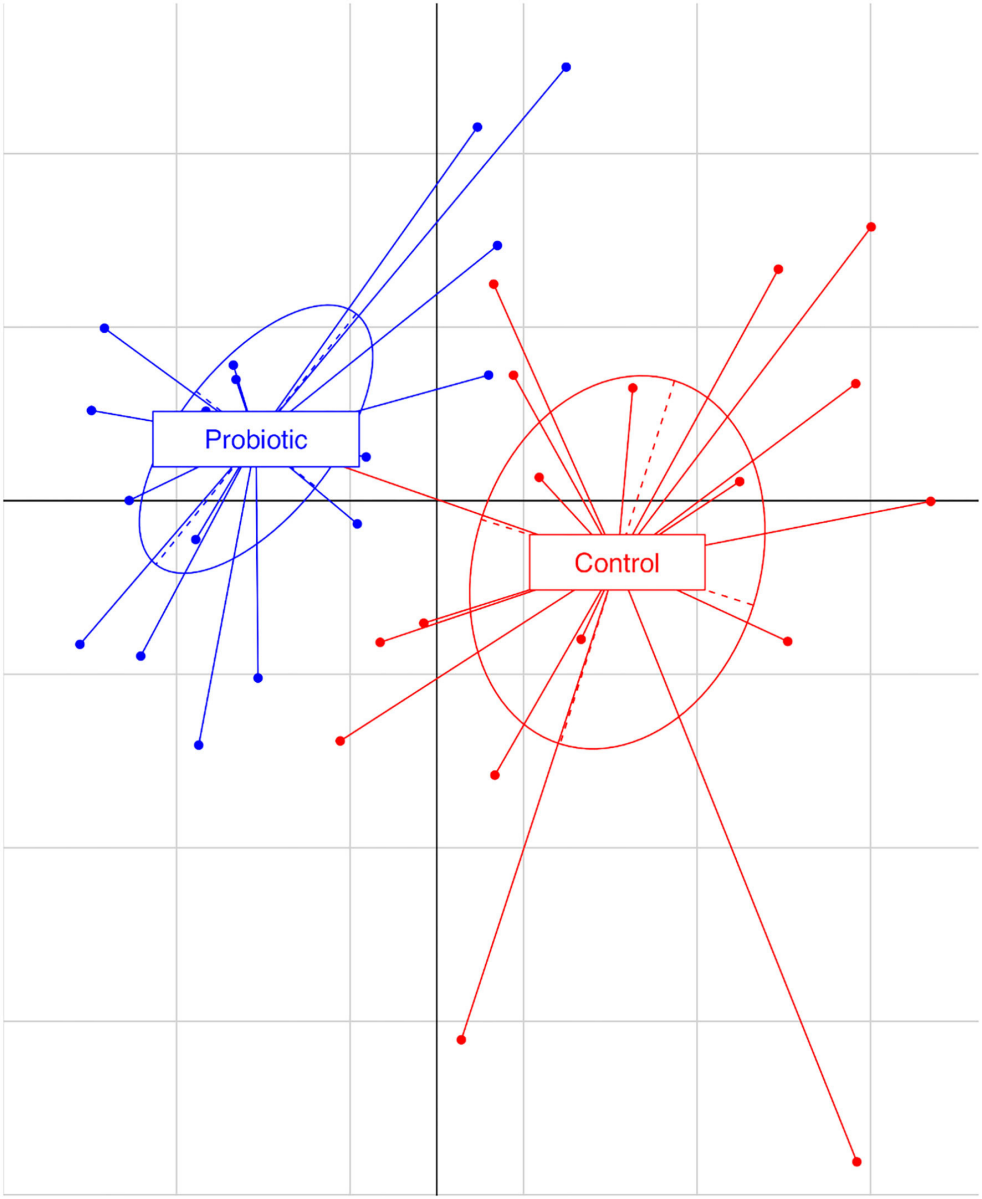
Principal component analysis based on observational taxonomic units (OTUs) relative abundance. The first component (horizontal) accounts for the 17.04% of the variance and the second component (vertical) accounts for the 12.73%. Blue dots represent the treated group (P), receiving water plus a commercial multi-strain probiotic (Slab51®), red dot represent the control group (C).

**Figure 3:**
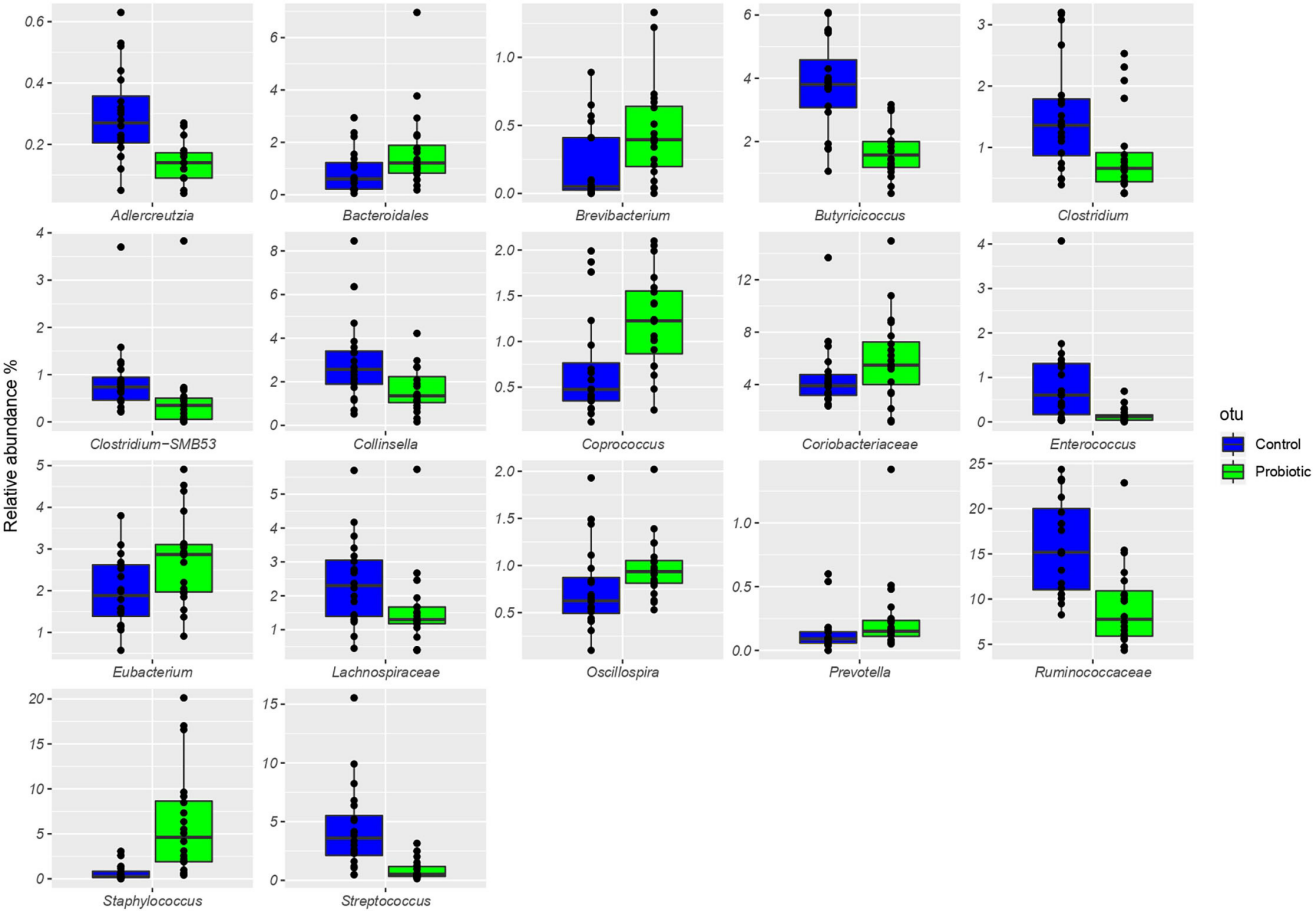
Boxplots showing the relative abundance at genus or family level of the OTUs differentially abundant based on Wilcoxon matched pairs test (*p* < 0.05) in cecal samples of the treated group (P), receiving water plus a commercial multi-strain probiotic Slab51® (blue bars), and the control group C (green bars).

**Figure 4:**
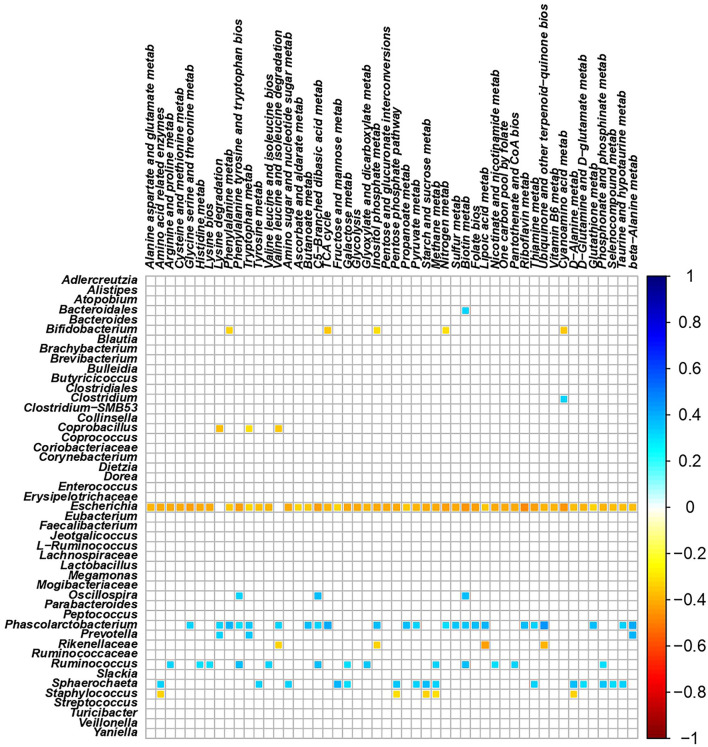
Correlation plot showing the Spearman's correlation between OTUs and predicted metabolic pathways. The intensity of the colors represents the degree of correlation between the OTUs and predicted metabolic pathways as measured by the Spearman's correlation. Only significant correlation (*p* < 0.05) are displayed.

The total number of high-quality paired-end sequences obtained from 16S rRNA sequencing reached 1,040,070 reads, with a median value of 15.997 ± 18.693 reads/sample, and a mean sequence length of 460 bp. The rarefaction analysis and Good's coverage, expressed as a median percentage (94%), indicated also satisfactory coverage. No significant differences were observed between the control and probiotic feeding diet in terms of complexity or number of OTU observed (Supplementary Table 1). The microbiota was dominated by the presence of *Ruminococcaceae* (present at 16 vs. 9% of the relative abundance in the C and P groups, respectively), *Lactobacillus* (10 vs. 8%) *Faecalibacterium* (8 vs. 7%), *Bacteroides* (7 vs. 8%), *Coriobacteriaceae* (5 vs. 6%), *Streptococcus* (4 vs. 1%), *Peptococcus*, and *Clostridiales* (4 vs. 3%). Going more deeply into the microbiota comparison, analysis of similarity (ANOSIM) statistical tests based on the OTU table showed significant differences among samples as a function of the probiotic (*p* < 0.05). Principal component analysis (Figure 2) showed a clear separation of the microbiota as a function of the dietary supplemention. ADONIS and ANOSIM statistical tests confirmed this differences (*p* < 0.05). In particular, we observed that probiotic inclusion enriched the presence of *Brevibacterium, Coprococcus, Eubacterium, Oscillospira, Prevotella*, and *Staphylococcus* genera. Instead, *Adlercreutzia, Clostridium, Collinsella, Enterococcus, Lachnospiraceae*, and *Streptococcus* were observed to be less abundant in birds with probiotic inclusion if compared with the control ones (Figure 3). Among *Ruminococcaceae*, they reduced in the probiotic group (*p* < 0.001), but this reduction did not affect the genus *Ruminococcus*, with its relative abundance not significantly different between the P and C groups (*p* = 0.879). Therefore, relative abundance of Ruminococcus among other Ruminococcaceae was higher in the P (7%) than in the C (4%) group. By plotting the correlation between OTU and inferred metabolic pathway (Figure 4) we observed a strong positive correlation between *Oscillospira* and *phenylalaline*, C5-branched and biotin metabolism, and a positive correlation between *Prevotella* with lysine and triptophane metabolism. In addition, a direct correlation between *Clostridium* and cyanoamino metabolism was observed. From the correlation analysis we also observed that *Ruminococcus* and *Phascolarctobacterium* showed the highest number of positive correlation while *Escherichia* showed the highest negative correlation.

## Discussion

This study demonstrated that a dietary probiotic complex containing *Streptococcus, Bifidobacterium*, and *Lactobacillus* strains improves histomorphometric characteristics of the guinea fowl (*Numida meleagris*) intestine and induces modification in cecum microbiota. In particular, the probiotic formulation employed in this study significantly increased the VW as well as the CD in the duodenum, ileum, and cecum of the guinea fowls. It is the opinion of the authors that the difference in VH observed in the cecum is not false positive because the villus height in that intestinal tract was highly variable (VH = 464.81 ± 232.47 in C, and VH = 594.97 ± 268.88 in P), and likely hid part of the probiotic effect. In addition, the difference was consistent with the increase in VH, which was observed in the other tracts of the intestine. These results are not consistent with those obtained with other probiotics in the intestine of other avian species. This is in line with the view that the probiotic administration effect depends on the treated avian species and its original gastrointestinal microbial background composition. For example, Protexin® (a multi-strain probiotic containing various bacterial and two yeast strains including *Lactobacillus plantarum, L. acidophilus, L. delbrueckii* subsp. *bulgaricus, Bifidobacterium bifidum, L. rhamnosus, Enterococcus faecium, Streptococcus salivarius* subsp. *thermophilus, Aspergillus oryza*, and *Candida pintolopesii*) induced an increase in the duodenal and ileal VH and CD in Japanese quail (15). The same probiotics increased VH, CD, and VH:CD ratio in the ileum of Ross broiler chickens (18), duodenal VH and VW as well as the ileal CD in the Leghorn Hy-line W36 white layer hens (36), and the duodenal VH and CD, unchanging ileal parameters, in the Cobb 500 broiler chicks (37). The latter birds showed an increase in VH in the duodenum and ileum when fed with a diet containing *L. salivarius* ssp. *salicinius* JCM 1230 and *L. agilis* JCM 1048 (13). No duodenal effect has been observed in the Hy-line laying hens receiving a diet supplemented with *Lactobacillus acidophilus* and *Bacillus subtilis* (38). However, *L. acidophilus* increased VW in the ileum of Kabir chicks (17). Dietary administration of the *B. subtilis* increased VH and VH:CD ratio in both the duodenum and ileum of Ross broilers (39), unchanging duodenal villi morphology, but increasing ileal VH and VH:CD ratio of common broiler chicks (40). Moreover, PrimaLac, a probiotics containing *L. casei, L. acidophilus, Bifidobacterium thermophilum*, and *Enterococcus faecium*, did not affect the histological characteristics of the small intestine of broiler chickens (41) and turkey poults (12). Lastly, a diet containing *Clostridium butyrricum* increased VH and VH:CD of the ileum and cecum from Lohmann pink laying hens (42). The increase in intestinal VH is related to the enhancement of absorptive surface area, for high expression of brush border enzymes capable of greater digestion, and absorption of available nutrients (15, 40, 41, 43, 44) improving growth performance (45). The multi-strain probiotic used in the present study contained lactic acid bacteria, which play a role in the intestinal increase of amylase level and VH (46, 47). Furthermore, we have to take into account that *Lactobacillus* dietary supplementation could increase VH even by means of volatile fatty acids, produced by carbohydrate digestion, which could nourish the intestinal villi leading to an enhancement of VH (37). Last, the increased VH observed in the guinea fowls from group P, compared with group C, may have been due to bacterial–diet interactions and the need for greater absorptive area to accommodate the byproducts associated with microbial fermentation (48). Similar to the VH, VW has been positively related with the absorptive efficiency of intestine in chickens and an increase in the diameter of VW is also closely related to an increase in the absorbent surface (49, 50). We have correlated the increase in both VH and VW in the P birds, the increase in the intestinal absorbent surface and the improvement in body weight, as demonstrated by other researchers (36). Thus, the significant increase in VW in all intestinal tracts in animals of group P represents a further morphological sign related to the enhancement of the absorptive capacity induced by the probiotic used in the current study. Intestinal crypts consist of several cell types, including pluripotent stem cells and cells that differentiate into mature cell lineages during migration along the crypts, such as absorptive cells (enterocytes) and mucin secretory GCs. Therefore, the length of villi is related to the proliferating rate in the crypts and the differentiating rate of villus epithelial cells (15, 51). Crypt lengthening may be mediated by a spectrum of local, immune, and neuro–humoral factors as well as probiotic dietary administration (52). In this study, we observed that the probiotic supplementation increased the CD in the duodenum, ileum and cecum. It has been reported that CD is directly representative of the intestinal environment and may be used as an indicator of intestinal health (13). It has been detected that the intestinal pathogen *Salmonella Typhimurium* induced reduction in the intestinal crypt depth of broiler chicks and this was correlated to reduction in absorptive capacity of the villus epithelium due to decreased replacement of enterocytes (53). Enhancement of CD has been reported in the duodenum and ileum of Japanese quail (15), in the ileum of Ross broiler chickens (18), and in the ileum, but not in the duodenum, of Leghorn Hy-line W36 white layer hens fed with the multi-strain Protexin (36). Increase in CD is not a regular effect of the probiotic-fed birds. No significant change in CD has been detected in the duodenum and ileum of the *Lactobacillus*-treated broilers (13), in the duodenum of Leghorn Hy-line W36 white layer hens fed with Protexin® (36), and in the cecum of Lohmann pink laying hens fed with a diet containing both *Saccharomyces boulardii* and *Pediococcus acidilactici* (42). These findings suggest that also for the CD, the morphological modification strictly depends on the type of probiotics and the bird species. As regard the VH:CD ratio, no differences between the C and P groups were noticed in this study. This finding is not consistent with the large number of reports in poultry dietary experiments in which VH and VH:CD ratio show the same trend, i.e., both increase (18, 37, 42) or both remain unvaried (12, 16, 38–41). A few studies on fowls report no change in VH:CD ratio when the increase in VH occurs (14, 36). It is worth to highlight that Bontempo et al. (54) did not observe a significant change in VH:CD ratio in piglets fed with dietary supplementation of *Saccharomyces cerevisiae* ssp. *boulardii* showing greater VH and CD than controls. Villus height and crypt depth represent an indirect indication of the maturity and functional capacity of enterocytes, and the longer the villi and crypts are, the greater number of enterocytes are there present (51). The unchanged VH:CD ratio in the group P of guinea fowls, when compared with C suggests that the higher VH observed in P may be due to their deeper crypts and that the used probiotic did not alter the balance between production and renewal of the cell types constituting the villi. The increase in VH is proportional with the deepening of the crypts, and that therefore, while keeping the constant VH:CD ratio with the C group, is even more evident than an increase in the absorbent surface. Despite the stimulation with Slab51 allowing the increase in intestinal absorbent surface in the P group compared with C, the VH:CD ratio remained unchanged, allowing proper bowel function. Although there is a simultaneous increase in the VH and CD parameters, there is no statistically significant increase in the GCs density (number of cells/100 μm of the villus) observed in all intestinal tracts of guinea fowls from the P group compared with group C. Same result has been reported for the ileum of Protexin-supplemented Ross broilers (18), although those probiotics induced an increase of the GCs density in small intestine of the Japanese quail (15). However, as it was to be expected, due to greater VH of the P animals, we counted a higher (*p* < 0.001) number of GCs/villus than in the intestine of the control ones. The presence of a greater number of GCs in the guinea fowls from the P group is an important fact, since these cells influence the quantity and quality of the mucus that covers the villi (55). Mucins perform a very important function in the modulation of the intestinal microbiota, as they contain a very large number of glycoproteic motifs of attack for bacteria, contributing to the mechanism of the non-immune exclusion of the intestinal microbiota (56). According to this mechanism, the different bacterial species can adhere to the mucins and not directly to the epithelial cells, and therefore be eliminated with the replacement of the mucins themselves. In return, the glycan repertoire of mucins can select for distinct mucosa-associated bacteria that are able to bind or degrade specific mucin glycans as a nutrient source (56). With regard to the high presence of GCs producing acidic glycans, this finding has also been reported in the intestine of other avians (37, 57). Acidic glycans are involved in regulating the interactions with microorganisms and parasitic helminths, as well as inpreventing inflammatory disorders (11, 58, 59). The negative charge of the acidic glycans can protect the host from mucin degradation by glycosidases (60–62), thereby impeding colonization by enteric pathogens and reducing the gastrointestinal infections (48, 63). Several factors such as hormones (neuropeptides), inflammatory mediators, and microbial colonization can affect the GCs activity and the secretion of mucin (19, 64). Bacterial colonization acts on mucin production and GCs proliferation *via* prostaglandin (65), cytokines (66), lipopolysaccharides (LPS) and flagellin A (from Gram-negative bacteria), and lipoteichoic acids (LTA) (from Gram-positive bacteria) (64). Like in mammals, and also in the chicken intestine, the probiotics have been demonstrated to positively modify mucin dynamics (14, 48, 67). This response to bacterial colonization is consistent with several reports attesting to the importance of the microbiota for the function and health of the bird intestine (12, 13, 16, 40, 42). High levels of diversity have been described as beneficial because it can improve microbiota stability and resilience facing environmental stresses (68), enhancing its resistance against invading pathogens (69). Although being well-documented in other species, studies in birds report conflicting results about the positive effect of higher level of diversity, not always associated with better productive performances (70). In poultry, several taxa can have high relevance on the intestinal health due to their specific metabolic features. Particularly *Ruminococcaceae*, which represent the most important taxon in both the probiotic and control groups, have been reported to have a key role in the gut physiology due to the production of short-chain fatty acids (SCFAs), vital energy source for enterocytes (71), and the degradation of cellulose (72). Moreover, *Coprococcus, Oscillospira*, and *Eubacterium* genera, which were found to be enriched by the probiotic administration, are known to produce butyrate (33, 73, 74) which exerts a beneficial effect on growth performance, structure of villi, and pathogen control, and has anti-inflammatory properties (75). In particular, we observed a strong association between *Oscillospira* and inferred metabolic pathways belonging to butanoate metabolism that confirms the beneficial role of this taxa in gut of the animals. Regarding *Coprococcus* and *Eubacterium*, both are considered as beneficial microbes able to increase cholesterol synthesis (via acetate), gluconeogenesis (via propionate), and energy source for colonocytes (via butyrate) (76). Our results show that in the intestine of the guinea fowls of group P, the treatment with probiotics simultaneously increased *Ruminococcaceae* and *Coprococcus*, suggesting a favorable ratio in the production of butyrate. In guinea fowls, the treatment induces a new bacterial asset, which is biochemically characterized by the coexistence of *Coprococcus* (acetate producer) and *Ruminococcaceae* (acetate consumers and butyrate producers). Those species are metabolically complementary and might lead to an increase in butyrate production. Butyrate-producing bacteria, as *Ruminococcaceae*, present in the GI tract of birds and mammals, can be net utilizers of acetate, which is normally regarded as an end-stage product of anaerobic fermentation (77). Also, the butyrate provides a fuel for epithelial cells of the large intestine thereby influencing colonic/cecal health, GCs differentiation, and mucin composition (56). Different studies demonstrated that *Ruminococcaceaeae, Faecalibacterium*, and *Roseburia* spp., grown in the presence of 60 mm acetate and 10 mm glucose, derived 85–90% butyrate-C from external acetate (77). This was due to the rapid interchange between extracellular acetate and intracellular acetyl-CoA, plus net acetate uptake. In contrast, a *Coprococcus*-related strain that is a net acetate producer derived only 28% butyrate-C from external acetate (77). Although we did not evaluate short-chain fatty acids in the feces, it should be noted that the probiotic administration enriches the presence of *Prevotella* that several studies reported to be considered as a taxon associated with a healthy gut due its ability to produce SCFAs (78, 79), improved glucose metabolism (80), or an overall anti-inflammatory effect (81). A positive effect on modulation of the cecal microbiota by the administration of the probiotic was also observed in the reduction of *Collinsella* reported as a pro-inflammatory taxon that can affect the metabolism by decreasing liver glycogenesis (82). The abundance of *Lachnospiraceae* was lower in the P group than in C. They have been associated to poor weight gain and higher feed conversion rate (FCR) in poultry because of the production of other SCFAs than butyrate, particularly propionate, which could induce satiety (83, 84) reducing food intake. However, their presence should not be considered negative since acetate, as previously mentioned, would promote the growth of *Bifidobacteria* (70). The abundance of *Lactobacillales* have been reported to decrease in chicken gut since the second week of age, being replaced by other *Firmicutes*, while their persistence has been related to lower performance. On the other hand, *Lactobacillus* producing lactic acid would cause a pH reduction which may inhibit pathogens (70). A few studies have described cecal microbiota of guinea fowls, but from the evidence on poultry, it was possible to determine that the dietary supplement of Slab51® probiotic increased microbiota diversity, and therefore more resilient to stress and more resistant to colonization by pathogenic organisms (70). Despite the administration of the probiotic mixture that was started when the birds were already 10 days old, due to the unavailability of 1-day old birds from the supplier, the increase of VH, VW, CD, and GCs and goblet cells per villus together with the enrichment of several beneficial taxa such as *Oscillospira, Eubacterium, Prevotella*, and some members of the *Lachnospiraceae* (e.g., *L-ruminococcus, Blautia*, and *Coprococcus*) provide evidence of the positive influence of this commercial multi-strain probiotic on the intestinal mucosal morphology and microbiota composition of guinea fowls.

## Data Availability Statement

The datasets presented in this study can be found in online repositories. The names of the repository/repositories and accession number(s) can be found below: https://www.ncbi.nlm.nih.gov/genbank/, PRJNA630376.

## Ethics Statement

The animal study was reviewed and approved by the Animal Welfare Body of the University of Camerino, Italy (prot. 4/2021).

## Author Contributions

LG, AR, LB, and GR were responsible for the conception of the study. SD, LG, GR, and AB performed data interpretation and wrote the manuscript. SD and NS performed morphological analysis. PR, PN, AB, AA, and IF analyzed the fecal microbiota. LG, LB, LF, AA, and GR reviewed the manuscript and provided critical suggestions and comments. All authors discussed the results and approved the final manuscript.

## Conflict of Interest

The authors declare that the research was conducted in the absence of any commercial or financial relationships that could be construed as a potential conflict of interest.

## Publisher's Note

All claims expressed in this article are solely those of the authors and do not necessarily represent those of their affiliated organizations, or those of the publisher, the editors and the reviewers. Any product that may be evaluated in this article, or claim that may be made by its manufacturer, is not guaranteed or endorsed by the publisher.

## References

[1] Ayasan T. Effects of dietary inclusion of Protexin (probiotic) on hatchability of Japanese quails. Indian J Anim Sci. (2013) 83:78–81.

[2] Patterson JA Burkholder KM. Application of prebiotics and probiotics in poultry production. Poult Sci. (2003) 82:627–31. 10.1093/ps/82.4.627 12710484

[3] Guarner F Schaafsma GJ. Probiotics. Int J Food Microbiol. (1998) 39:237–8. 10.1016/S0168-1605(97)00136-0 9553803

[4] Fuller R. Reasons for the apparent variation in the probiotic response. Biologia. (2006) 61:751–4. 10.2478/s11756-006-0152-3

[5] Meng QW Yan L Ao X Zhou TX Wang JP Lee JH. Influence of probiotics in different energy and nutrient density diets on growth performance, nutrient digestibility, meat quality, and blood characteristics in growing-finishing pigs. J Anim Sci. (2010) 88:3320–6. 10.2527/jas.2009-2308 20562363

[6] Dunne C O'Mahony L Murphy L Thornton G Morrisey D O'Halloran S Feeney M. *In vitro* selection criteria for probiotic bacteria of human origin: correlation with *in vivo* findings. Am J Clin Nutr. (2001) 73:386S–92S. 10.1093/ajcn/73.2.386s 11157346

[7] Delgado S O'Sullivan E Fitzgerald G Mayo B. *In vitro* evaluation of the probiotic properties of human intestinal Bifidobacterium species and selection of new probiotic candidates. J Appl Microbiol. (2008) 104:1119–27. 10.1111/j.1365-2672.2007.03642.x 18248372

[8] Sandra A dos Reis LL da Conceição NP Siqueira DD Rosa LL da Silva M. Review of the mechanisms of probiotic actions in the prevention of colorectal cancer. Nutr Res. (2017) 37:1–19. 10.1016/j.nutres.2016.11.009 28215310

[9] Uriot O Denis S Junjua M Roussel Y Dary-Mourot A Blanquet-Diot S. *Streptococcus thermophilus*: from yogurt starter to a new promising probiotic candidate? J. Funct Foods. (2017) 37:74–89. 10.1016/j.jff.2017.07.038

[10] Chapman CMC Gibson GR Rowland I. *In vitro* evaluation of single- and multi-strain probiotics: inter-species inhibition between probiotic strains, and inhibition of pathogens. Anaerobe. (2012) 18:405–13. 10.1016/j.anaerobe.2012.05.004 22677262

[11] Ouwerkerk JP de Vos WM Belzer C. Glycobiome: bacteria and mucus at the epithelial interface. Best Pract Res Clin Gastroenterol. (2013) 27:25–38. 10.1016/j.bpg.2013.03.001 23768550

[12] Rahimi S Grimes JL Fletcher O Oviedo E Sheldon BW. Effect of a direct-fed microbial (Primalac) on structure and ultrastructure of small intestine in turkey poults. Poult Sci. (2009) 88:491–503. 10.3382/ps.2008-00272 19211517

[13] Meimandipour A Hair-Bejo M Shuhaimi M Azhar K Soleimani AF Rasti B. Gastrointestinal tract morphological alteration by unpleasant physical treatment and modulating role of *Lactobacillus* in broilers. Br Poult Sci. (2010) 51:52–9. 10.1080/00071660903394455 20390569

[14] Aliakbarpour HR Chamani M Rahimi G Sadeghi AA Qujeq D. The *Bacillus subtilis* and lactic acid bacteria probiotics influences intestinal mucin gene expression, histomorphology and growth performance in broilers. Asian-Aust J Anim Sci. (2012) 25:1285–93. 10.5713/ajas.2012.12110 25049692 PMC4092943

[15] Seifi K Karimi Torshizi MA Rahimi S Kazemifard M. Efficiency of early, single-dose probiotic administration methods on performance, small intestinal morphology, blood biochemistry, and immune response of Japanese quail. Poult Sci. (2017) 96:2151–8. 10.3382/ps/pew446 28521051

[16] Deng W Dong XF Tong JM Zhang Q. The probiotic Bacillus licheniformis ameliorates heat stress-induced impairment of egg production, gut morphology, and intestinal mucosal immunity in laying hens. Poult Sci. (2012) 91:575–82. 10.3382/ps.2010-01293 22334732

[17] Forte C Manuali E Abbate Y Papa P Vieceli L Tentellini M. Dietary *Lactobacillus acidophilus* positively influences growth performance, gut morphology, and gut microbiology in rurally reared chickens. Poult Sci. (2018) 97:930–6. 10.3382/ps/pex396 29294082 PMC5850662

[18] Kazemi SA Ahmadi H Karimi Torshizi MA. Evaluating two multistrain probiotics on growth performance, intestinal morphology, lipid oxidation and ileal microflora in chickens. J Anim Physiol Anim Nutr. (2019) 103:1399–407. 10.1111/jpn.13124 31141245

[19] Kim YS Ho SB. Intestinal goblet cells and mucins in health and disease: recent insights and progress. Curr Gastroenterol Rep. (2010) 12:319–30. 10.1007/s11894-010-0131-2 20703838 PMC2933006

[20] Forstner JF Forstner GG. Gastrointestinal mucus. In: Johnson LR, editor. Physiology of the Gastrointestinal Tract. New York, NY: Raven Press (1994). p. 1245–83.

[21] Liévin-Le Moal V Servin AL. The front line of enteric host defense against unwelcome intrusion of harmful microorganisms: mucins, antimicrobial peptides, and microbiota. Clin Microbiol Rev. (2006) 19:315–37. 10.1128/CMR.19.2.315-337.2006 16614252 PMC1471992

[22] Nahashon SN Aggrey SE Adefope NA Amenyenu A Wright D. Growth characteristics of pearl gray guinea fowl as predicted by the Richards, Gompertz, and logistic models. Poult Sci. (2006) 85:359–63. 10.1093/ps/85.2.359 16523639

[23] Gholipour V Chamani M Shahryar HA Sadeghi A Aminafshar M. Effects of dietary L-glutamine supplement on performance, characteristics of the carcase and intestinal morphometry in guinea fowl chickens (*Numida meleagris*). Ital J Anim Sci. (2019) 18:513–21. 10.1080/1828051X.2018.1544856

[24] Accogli G Crovace A Mastrodonato M Rossi G Francioso EG Desantis S. Probiotic supplementation affects the glycan composition of mucins secreted by Brunner's glands of the pig duodenum. Ann Anat. (2018) 218:236–42. 10.1016/j.aanat.2018.03.008 29730471

[25] Desantis S Mastrodonato M Accogli G Rossi G Crovace AM. Effects of a probiotic on the morphology and mucin composition of pig intestine. Histol Histopathol. (2019) 34:1037–50. 10.14670/HH-18-106 30916355

[26] Rossi G Pengo G Caldin M Palumbo Piccionello A Steiner JM Cohen ND. Comparison of microbiological, histological, and immunomodulatory parameters in response to treatment with either combination therapy with prednisone and metronidazole or probiotic VSL#3 strains in dogs with idiopathic inflammatory bowel disease. PLoS One. (2014) 9:e94699. 10.1371/journal.pone.0094699 24722235 PMC3983225

[27] Rossi G Jergens A Cerquetella M Berardi S Di Cicco E Bassotti G. Effects of a probiotic (SLAB51™) on clinical and histologic variables and microbiota of cats with chronic constipation/megacolon: a pilot study. Benef Microbes. (2018) 9:101–10. 10.3920/BM2017.0023 29065705

[28] Guyonnet D Woodcock A Stefani B Trevisan C Hall C. Fermented milk containing *Bifidobacterium lactis* DN-173 010 improved self-reported digestive comfort amongst a general population of adults. A randomized, open-label, controlled, pilot study. J Dig Dis. (2009) 10:61–70. 10.1111/j.1751-2980.2008.00366.x 19236549

[29] Mowry RW. The special value of methods that color both acidic and vicinal hydroxyl groups in the histochemical study of mucins. With revised directions for the colloidal iron stain, the use of Alcian blue G8X and their combinations with the periodic acid-Schiff reaction. Ann N Y Acad Sci. (1967) 106:402–23. 10.1111/j.1749-6632.1963.tb16654.x

[30] Klindworth A Pruesse E Schweer T Peplies J Quast C Horn M. Evaluation of general 16S ribosomal RNA gene PCR primers for classical and next-generation sequencing-based diversity studies. Nucleic Acids Res. (2013) 41:e1. 10.1093/nar/gks808 22933715 PMC3592464

[31] Faul F Erdfelder E Lang AG Buchner A. G*Power 3: A flexible statistical power analysis program for the social, behavioral, and biomedical sciences. Behav Res Methods. (2007) 39:175–91. 10.3758/BF03193146 17695343

[32] Storey JD. The positive false discovery rate: a Bayesian interpretation and the q-value. Ann Statist. (2003) 31:2013–35. 10.1214/aos/1074290335

[33] Biasato I Ferrocino I Biasibetti E Grego E Dabbou S Sereno A. Modulation of intestinal microbiota, morphology and mucin composition by dietary insect meal inclusion in free-range chickens. BMC Vet. Res. (2018) 14:383. 10.1186/s12917-018-1690-y 30514391 PMC6278000

[34] Dixon P. VEGAN, a package of R functions for community ecology. J Veg Sci. (2003) 14:927–30. 10.1111/j.1654-1103.2003.tb02228.x

[35] Langille MG Zaneveld J Caporaso JG McDonald D Knights D Reyes JA. Predictive functional profiling of microbial communities using 16S rRNA marker gene sequences. Nat Biotechnol. (2013) 31:814–21. 10.1038/nbt.2676 23975157 PMC3819121

[36] Shalaei M Hosseini SM Zergani E. Effect of different supplements on eggshell quality, some characteristics of gastrointestinal tract and performance of laying hens. Vet Res Forum. (2014) 5:277–86. 25610579 PMC4299993

[37] Shah M Zaneb H Masood S Khan RU Ashraf S Sikandar A. Effect of dietary supplementation of zinc and multi-microbe probiotic on growth traits and alteration of intestinal architecture in broiler. Probiotics Antimicrob Proteins. (2019) 11:931–7. 10.1007/s12602-018-9424-9 29680883

[38] Forte C Acuti G Manuali E Casagrande Proietti P Pavone S Trabalza-Marinucci M. Effects of two different probiotics on microflora, morphology, and morphometry of gut in organic laying hens. Poult Sci. (2016) 95:2528–35. 10.3382/ps/pew164 27143778

[39] Sen S Ingale SL Kim YW Kim JS Kim KH Lohakare JD. Effect of supplementation of Bacillus subtilis LS 1-2 to broiler diets on growth performance, nutrient retention, caecal microbiology and small intestinal morphology. Res Vet Sci. (2012) 93:264–8. 10.1016/j.rvsc.2011.05.021 21757212

[40] Ma Y Wang W Zhang H Wang J Zhang W Gao J. Supplemental *Bacillus subtilis* DSM 32315 manipulates intestinal structure and microbial composition in broiler chickens. Sci Rep. (2018) 8:1–13. 10.1038/s41598-018-33762-8 30337568 PMC6194052

[41] Chichlowski M Croom WJ Edens FW McBride BW Qiu R Chiang CC. Microarchitecture and spatial relationship between bacteria and ileal, cecal, and colonic epithelium in chicks fed a direct-fed microbial, PrimaLac, and salinomycin. Poult Sci. (2007) 86:1121–32. 10.1093/ps/86.6.1121 17495082

[42] Xiang Q Wang C Zhang H Lai W Wei H Peng J. Effects of different probiotics on laying performance, egg quality, oxidative status, and gut health in laying hens. Animals (Basel). (2019) 9:1110. 10.3390/ani9121110 31835513 PMC6940752

[43] Pluske JR Thompson MJ Atwood CS Bird PH Williams IH Hartmann PE. Maintenance of villus height and crypt depth, and enhancement of disaccharide digestion and monosaccharide absorption, in piglets fed on cows' whole milk after weaning. Br J Nutr. (1996) 76:409–22. 10.1079/BJN19960046 8881713

[44] Awad W Ghareeb K Abdel-Raheem S Böhm J. Effects of dietary inclusion of probiotic and synbiotic on growth performance, organ weights, and intestinal histomorphology of broiler chickens. Poult Sci. (2009) 88:49–56. 10.3382/ps.2008-00244 19096056

[45] Rezaei M Karimi Torshizi MA Wall H Ivarsson E. Body growth, intestinal morphology and microflora of quail on diets supplemented with micronised wheat fibre. Br Poult Sci. (2018) 59:422–9. 10.1080/00071668.2018.1460461 29620417

[46] Jin LZ Ho YW Abdullah N Jalaludin S. Digestive and bacterial enzyme activities in broilers fed diets supplemented with Lactobacillus cultures. Poult Sci. (2000) 79:886–91. 10.1093/ps/79.6.886 10875772

[47] Gutierrez-Fuentes C Zuniga-Orozco LA Vicente JL Hernandez-Velasco X Menconi A Kuttapan VA. Effect of a lactic acid bacteria based probiotic, Floramax-B11®, on performance, bone qualities, and morphometric analysis of broiler chickens: an economic analysis. Int J Poult Sci. (2013) 12:322–7. 10.3923/ijps.2013.322.327

[48] Forder RE Howarth GS Tivey DR Hughes RJ. Bacterial modulation of small intestinal goblet cells and mucin composition during early posthatch development of poultry. Poult Sci. (2007) 86:2396–403. 10.3382/ps.2007-00222 17954591

[49] Sun Y Rajput IR Arain MA Li Y Baloch DM. Oral administration of *Saccharomyces boulardii* alters duodenal morphology, enzymatic activity and cytokine production response in broiler chickens. Anim Sci J. (2017) 88:1204–11. 10.1111/asj.12757 27925366

[50] Wang F Zuo Z Chen K Gao C Yang Z Zhao S. Histopathological injuries, ultrastructural changes, and depressed TLR expression in the small intestine of broiler chickens with aflatoxin B1. Toxins (Basel). (2018) 10:131. 10.3390/toxins10040131 29561786 PMC5923297

[51] Hampson DJ. Alterations in piglet small intestinal structure at weaning. Res Vet Sci. (1986) 40:32–40. 10.1016/S0034-5288(18)30482-X 3704321

[52] Pearson JP Brownlee IA. The interaction of large bowel microflora with the colonic mucus barrier. Int J Inflam. (2010) 2010:321426. 10.4061/2010/321426 21152122 PMC2989700

[53] Fasina YO Hoerr FJ McKee SR Conner DE. Influence of Salmonella enterica serovar Typhimurium infection on intestinal goblet cells and villous morphology in broiler chicks. Avian Dis. (2010) 54:841–7. 10.1637/9055-090809-Reg.1 20608528

[54] Bontempo V Di Giancamillo A Savoini GV Dell'Orto V Domeneghini C. Live yeast dietary supplementation acts upon intestinal morpho-functional aspects and growth in weanling piglets. Anim Feed Sci Technol. (2006) 129:224–36. 10.1016/j.anifeedsci.2005.12.015

[55] Wrzosek L Miquel S Noordine ML Bouet S Joncquel Chevalier-Curt M Robert V. Bacteroides thetaiotaomicron and Faecalibacterium prausnitzii influence the production of mucus glycans and the development of goblet cells in the colonic epithelium of a gnotobiotic model rodent. BMC Biol. (2013) 11:61. 10.1186/1741-7007-11-61 23692866 PMC3673873

[56] Schroeder BO. Fight them or feed them: how the intestinal mucus layer manages the gut microbiota. Gastroenterol Rep. (2019) 7:3–12. 10.1093/gastro/goy052 30792861 PMC6375348

[57] Ashraf S Zaneb H Yousaf MS Ijaz A Sohail MU Muti S. Effect of dietary supplementation of prebiotics and probiotics on intestinal microarchitecture in broilers reared under cyclic heat stress. J Anim Physiol Anim Nutr. (2013) 97:68–73. 10.1111/jpn.12041 23639019

[58] Kawashima H. Roles of sulfated glycans in lymphocyte homing. Biol Pharm Bull. (2006) 29:2343–9. 10.1248/bpb.29.2343 17142960

[59] Sharpe C Thornton DJ Grencis RK. A sticky end for gastrointestinal helminths; the role of the mucus barrier. Parasite Immunol. (2018) 40:e12517. 10.1111/pim.12517 29355990 PMC5900928

[60] Corfield AP Wagner SA Clamp JR Kriaris MS Hoskins LC. Mucin degradation in the human colon: production of sialidase, sialateO-acetylesterase, N-acetylneuraminatelyase, arylesterase, and glycosulfatase activities by strains of fecal bacteria. Infect Immun. (1992) 60:3971–8. 10.1128/iai.60.10.3971-3978.1992 1398908 PMC257425

[61] Kamisago S Iwamori M Tai T Mitamura K Yazaki Y Sugano K. Role of sulfatides in adhesion of *Helicobacter pylori* to gastric cancer cells. Infect Immun. (1996) 64:624–8. 10.1128/iai.64.2.624-628.1996 8550217 PMC173811

[62] Veerman EC Bank CM Namavar F Appelmelk BJ Bolscher JG NieuwAmerongen AV. Sulfated glycans on oral mucin as receptors for *Helicobacter pylori*. Glycobiol. (1997) 7:737–43. 10.1093/glycob/7.6.737 9376676

[63] Struwe WB Gough R Gallagher ME Kenny DT Carrington SD Karlsson NG. Identification of O-glycan structures from chicken intestinal mucins provides insight into *Campylobacter jejuni* pathogenicity. Mol Cell Proteom. (2015) 14:1464–77. 10.1074/mcp.M114.044867 25776888 PMC4458713

[64] Dharmani P Srivastava V Kissoon-Singh V Chadee K. Role of intestinal mucins in innate host defense mechanisms against pathogens. J Innate Immun. (2009) 1:123–35. 10.1159/000163037 20375571 PMC7312850

[65] Kunikata T Tanaka A Miyazawa T Kato S Takeuchi K. 16,16-Dimethyl prostaglandin E2 inhibits indomethacin-induced small intestinal lesions through EP3 and EP4 receptors. Dig Dis Sci. (2002) 47:894–904. 10.1023/A:1014725024519 11991626

[66] Blanchard C Durual S Estienne M Bouzakri K Heim MH Blin N. IL-4 and IL-13 up-regulate intestinal trefoil factor expression: requirement for STAT6 and *de novo* protein synthesis. J Immunol. (2004) 172:3775–83. 10.4049/jimmunol.172.6.3775 15004182

[67] Smirnov A Perez R Amit-Romach E Sklan D Uni Z. Mucin dynamics and microbial populations in chicken small intestine are changed by dietary probiotic and antibiotic growth promoter supplementation. J Nutr. (2005) 135:187–92. 10.1093/jn/135.2.187 15671211

[68] Xue Z Zhang W Wang L Hou R Zhang M Fei L. The bamboo-eating giant panda harbors a carnivore-like gut microbiota, with excessive seasonal variations. MBio. (2015) 6:e00022–e15. 10.1128/mBio.00022-15 25991678 PMC4442137

[69] Kuhn I Katouli M Lund A Wallgren P Mollby R. Phenotype diversity and stability of intestinal coliform flora in piglets during the first three months of age. Microbial Ecol Health Dis. (1993) 6:101–7. 10.3109/08910609309141313

[70] Díaz-Sánchez S Perrotta AR Rockafellow I Alm EJ Okimoto R Hawken R. Using fecal microbiota as biomarkers for predictions of performance in the selective breeding process of pedigree broiler breeders. PLoS One. (2019) 14:e0216080. 10.1371/journal.pone.0216080 31063485 PMC6504170

[71] Sunkara LT Jiang W Zhang G. Modulation of antimicrobial host defense peptide gene expression by free fatty acids. PLoS One. (2012) 7:e49558. 10.1371/journal.pone.0049558 23166711 PMC3499459

[72] Liu C Finegold SM Song Y Lawson PA. Reclassification of *Clostridium coccoides, Ruminococcus hansenii, Ruminococcus hydrogenotrophicus, Ruminococcus luti, Ruminococcus productus* and *Ruminococcus schinkii* as *Blautia coccoides* gen. nov, comb nov, *Blautia hansenii* comb nov, *Blautia hydrogenotrophica* comb nov, *Blautia luti* comb nov, *Blautia producta* comb nov, *Blautia schinkii* comb nov and description of *Blautia wexlerae* sp nov, isolated from human faeces. Int J Syst Evol Microbiol. (2008) 58:1896–902. 10.1099/ijs.0.65208-0 18676476

[73] Pryde SE Duncan SH Hold GL Stewart CS Flint HJ. The microbiology of butyrate formation in the human colon. FEMS Microbiol Lett. (2002) 217:133–9. 10.1111/j.1574-6968.2002.tb11467.x 12480096

[74] Yang L Liu S Ding J Dai R He C Xu K. Gut microbiota co-microevolution with selection for host humoral immunity. Front Microbiol. (2017) 8:1243. 10.3389/fmicb.2017.01243 28725219 PMC5495859

[75] Onrust L Ducatelle R Van Driessche K De Maesschalck C Vermeulen K Haesebrouck F. (2015). Steering endogenous butyrate production in the intestinal tract of broilers as a tool to improve gut health. Front Vet Sci. 2:75. 10.3389/fvets.2015.00075 26734618 PMC4682374

[76] Vernocchi P Del Chierico F Putignani L. Gut microbiota profiling: metabolomics based approach to unravel compounds affecting human health. Front Microbiol. (2016) 7:1144. 10.3389/fmicb.2016.01144 27507964 PMC4960240

[77] Duncan SH Holtrop G Lobley GE Calder AG Stewart CS Flint HJ. Contribution of acetate to butyrate formation by human faecal bacteria. Br J Nutr. (2004) 91:915–23. 10.1079/BJN20041150 15182395

[78] De Filippo C Cavalieri D Di Paola M Ramazzotti M Poullet JB Massart S. Impact of diet in shaping gut microbiota revealed by a comparative study in children from Europe and rural Africa. Proc Natl Acad Sci U S A. (2010) 107:14691–6. 10.1073/pnas.1005963107 20679230 PMC2930426

[79] De Filippis F Pellegrini N Vannini L Jeffery IB La Storia A Laghi L. High-level adherence to a Mediterranean diet beneficially impacts the gut microbiota and associated metabolome. Gut. (2016) 65:1812–21. 10.1136/gutjnl-2015-309957 26416813

[80] De Vadder F Kovatcheva-Datchary P Zitoun C Duchampt A Bäckhed F Mithieux G. Microbiota-produced succinate improves glucose homeostasis via intestinal gluconeogenesis. Cell Metab. (2016) 24:151–7. 10.1016/j.cmet.2016.06.013 27411015

[81] De Angelis M Montemurno E Vannini L Cosola C Cavallo N Gozzi G. Effect of whole-grain barley on the human fecal microbiota and metabolome. Appl Environ Microbiol. (2015) 81:7945–56. 10.1128/AEM.02507-15 26386056 PMC4616929

[82] Gomez-Arango LF Barrett HL Wilkinson SA Callaway LK McIntyre HD Morrison M. Low dietary fiber intake increases Collinsella abundance in the gut microbiota of overweight and obese pregnant women. Gut Microbes. (2018) 9:189–201. 10.1080/19490976.2017.1406584 29144833 PMC6219589

[83] Lin HV Frassetto A Kowalik EJ Jr Nawrocki AR Lu MM Kosinski JR. Butyrate and propionate protect against diet-induced obesity and regulate gut hormones via free fatty acid receptor 3-independent mechanisms. PLoS One. (2012) 7:e35240. 10.1371/journal.pone.0035240 22506074 PMC3323649

[84] Reichardt N Duncan SH Young P Belenguer A McWilliam Leitch C Scott KP. Phylogenetic distribution of three pathways for propionate production within the human gut microbiota. ISME J. (2014) 8:1323–35. 10.1038/ismej.2014.14 24553467 PMC4030238

